# Linking brain structure to stress reactivity: cingulate surface area predicts acute cortisol responses – CORRIGENDUM

**DOI:** 10.1017/S0033291726104395

**Published:** 2026-04-28

**Authors:** Emin Serin, Lea Sophie Schill, Christoph Bärtl, Marina Giglberger, Julian Konzok, Hannah L. Peter, Nina Speicher, Ludwig Kreuzpointner, Brigitte M. Kudielka, Stefan Wüst, Henrik Walter, Gina-Isabelle Henze

**Affiliations:** 1 Department of Psychiatry and Neurosciences CCM, Charité – Universitätsmedizin Berlin, Corporate Member of Freie Universität Berlin and Humboldt-Universität zu Berlin, Berlin, Germany; 2 German Center for Mental Health (DZPG), Partner Site Berlin-Potsdam, Berlin, Germany; 3 Institute of Psychology, University of Regensburg, Regensburg, Germany; 4 Department of Epidemiology and Preventive Medicine, University of Regensburg, Regensburg, Germany

This article was originally published with incorrect affiliations for Emin Serin, Lea Sophie Schill, Christoph Bärtl, Henrik Walter, and Gina-Isabelle Henze.

The affiliation was originally given as Department of Psychiatry and Neurosciences CCM, Charité - Universitätsmedizin Berlin, corporate Member of Freie Universität Berl. The correct affiliation is Department of Psychiatry and Neurosciences CCM, Charité - Universitätsmedizin Berlin, Corporate Member of Freie Universität Berlin and Humboldt-Universität zu Berlin.

The errors have been corrected and the online HTML and PDF versions updated.
