# Supplementary material for: Linking brain structure to stress reactivity: cingulate surface area predicts acute cortisol responses
Source: Psychol Med. 2026 Apr 7;56:e96. doi: 10.1017/S0033291726104103 (PMC13079226; doi:10.1017/S0033291726104103)
Supplement: Serin et al. supplementary material [file S0033291726104103sup001.pdf]

Linking brain structure to stress reactivity: Cingulate surface area predicts acute cortisol responses

Emin Serin[1,2], Lea Sophie Schill[1], Christoph Bärtl[1,2], Marina Giglberger[3], Julian Konzok[3,4], Hannah L. Peter[3], Nina Speicher[3], Ludwig Kreuzpointner[3], Brigitte M. Kudielka[3], Stefan Wüst[3], Henrik Walter[1,2] & Gina-Isabelle Henze[1,2,*]

[1]Research Division of Mind and Brain, Department of Psychiatry and Psychotherapy CCM, Charité-Universitätsmedizin Berlin, Corporate Member of Freie Universität Berlin, Humboldt-Universität zu Berlin, and Berlin Institute of Health, Berlin, Germany

[2]German Center for Mental Health (DZPG), Partner Site Berlin - Potsdam, Germany

[3]Institute of Psychology, University of Regensburg, Regensburg, Germany

[4]Department of Epidemiology and Preventive Medicine, University of Regensburg, Regensburg, Germany

*Corresponding author email: gina-isabelle.henze@charite.de

Supplementary Material

Supplementary Methods

Statistical model tested with PALM:

Contrast	Male cortisol	Female cortisol	Cycle	Age	TBV*
Cortisol	1	1	0	0	0
Male	1	0	0	0	0
Female	0	1	0	0	0
Male > Female	1	-1	0	0	0
Female > Male	-1	1	0	0	0

*Total Brain Volume (TBV) was only included in volumetric models.

Feature Importance Analysis: Shapley additive explanations (SHAP) were utilized to identify contributing brain measures that drive cortisol increase prediction (Lundberg & Lee, 2017). SHAP is a model-agnostic method that measures the marginal contribution of each feature to a model prediction, providing a straightforward way to rank and visualize features based on their contribution. As ridge regression was employed, the linear SHAP estimator was used. We calculated SHAP values in two ways. First, after model training and evaluation, we fit the model using the entire sample and estimated SHAP values. This provides a single set of straightforwardly interpretable SHAP values (Figure 3). Second, we estimated SHAP values on the test set of each outer cross-validation fold, resulting SHAP values with a size of S x F in each fold, where S represents subjects within test fold and F represents brain measures. Within each fold, we took median absolute SHAP value for each brain measure, providing a final matrix of CV folds x brain measures. This allows us to evaluate the generalizability and variability of brain measure importance (Supplementary Figure 1). We identified important features based on the mean absolute SHAP value in each analysis and reported the top 20 informative brain measures.

Supplementary Tables

Table S1. Post-hoc comparisons of cortisol increase among sex and cycle groups.

Groups		Mean Difference	p
Males	Luteal Phase	-2.891	.001
Males	Contraceptive	-2.206	.001
Males	Post-Menopause	-2.150	.167
Luteal Phase	Contraceptive	.685	.637
Luteal Phase	Post-Menopause	.741	.900
Contraceptive	Post-Menopause	.056	1.0

One-Way ANOVA: $F(3, 287) = 13.70, p < .001)$. Post-hoc comparisons were done using Tukey's HSD.

Table S2. Sample characteristics of cortisol increase and total brain volume by sex.

	Mean	*SD*	*t*	*p*
Cortisol increase (nmol/L)				
Female	1.692	2.777	-6.291	.001
Male	4.248	4.108		
Total brain volume (mm^3)				
Female	1084907.554	77667.106	-14.226	.001
Male	1219128.567	83121.059		

Table S3. Significant clusters based on vertex-wise analysis of cortisol increase x structural brain measures effect.

Region	Measure	Contrast	Max. Vert. Sig. -log(*p*)	*p*	Cluster Size (mm^2)	MNI Coordinates (x, y, z)
Left cACC	Volume	Male	-4.855	.004	326.73	-8.2, 31.1, 20.3
Left cACC	Area	Male	-4.522	.007	458.99	-5.9, 24.3, 18.0

Max. Vert. Sig., maximum vertex significance; MNI, Montreal Neurological Institute

Table S4. Results of PALM calculations for cortical surface area of the left hemisphere.

Contrast		rACC	cACC	PCC	Parahippocampus	lOFC	mOFC	Insula	Precuneus
**Cortisol**									
	T	**-2.439**	**-3.415**	**-2.538**	-1.772	-1.110	-.282	**-2.508**	-1.097
	p_{uncorr}	**.015**	**.000**	**.010**	.075	.263	.783	**.014**	.273
	p_{FDR}	**.030**	**.003**	**.030**	.120	.312	.783	**.030**	.312
	d	**-.998**	**-1.398**	**-1.039**	-.726	-.454	-.116	**-1.027**	-.449
**Male**									
	t	**-2.655**	**-4.365**	**-2.374**	-1.020	-.601	-.043	-1.890	-.745
	p_{uncorr}	**.009**	**.000**	**.021**	.316	.555	.967	.060	.458
	p_{FDR}	**.038**	**.001**	.055	.505	.634	.967	.120	.611
	d	**-1.281**	**-2.106**	-1.146	-.492	-.290	-.021	-.912	-.359
**Female**									
	t	-1.084	-1.051	-1.409	-1.445	-.933	-.317	-1.723	-.812
	p_{uncorr}	.284	.290	.157	.151	.356	.752	.084	.414
	p_{FDR}	.465	.465	.418	.418	.473	.752	.418	.473
	d	-.543	-.526	-.705	-.723	-.467	-.159	-.862	-.407
**Male > Female**									
	t	-.675	-1.701	-.248	.573	.403	.232	.289	.222
	p_{uncorr}	.507	.087	.810	.569	.694	.817	.769	.819
	p_{FDR}	.819	.698	.819	.819	.819	.819	.819	.819
	d	-.322	-.812	-.118	.274	.193	.111	.138	.106
**Female > Male**									
	t	.675	1.701	.248	-.573	-.403	-.232	-.289	-.222
	p_{uncorr}	.507	.087	.810	.569	.694	.817	.769	.819
	p_{FDR}	.819	.698	.819	.819	.819	.819	.819	.819
	d	.322	.812	.118	-.274	-.193	-.111	-.138	-.106

p_{uncorr}: uncorrected *p*-values; p_{FDR}: FDR-corrected *p*-values; *d*: Cohen's *d*; rACC: rostral anterior cingulate cortex; cACC: caudal ACC; PCC: posterior cingulate cortex; lOFC: lateral orbitofrontal cortex; mOFC: medial OFC. Cortisol contrast represents a sex-independent cortisol increase using the whole sample. Significant results are given in bold.

Table S5. Results of PALM calculations for cortical surface area of the right hemisphere.

Contrast		rACC	cACC	PCC	Parahippocampus	lOFC	mOFC	Insula	Precuneus
**Cortisol**									
	t	-1.165	**-2.138**	-.887	-.984	-1.835	-1.829	-1.881	-1.4905
	p_{uncorr}	.254	**.033**	.368	.325	.067	.067	.056	.138
	p_{FDR}	.338	.134	.368	.368	.134	.134	.134	.221
	d	-.477	-.875	-.363	-.403	-.751	-.749	-.770	-.610
**Male**									
	t	-1.222	**-2.366**	-.902	-.509	-1.659	-1.214	-1.082	-.609
	p_{uncorr}	.229	**.020**	.376	.617	.104	.227	.279	.541
	p_{FDR}	.446	.158	.501	.617	.416	.446	.446	.617
	d	-.590	-1.142	-.435	-.245	-.800	-.586	-.522	-.294
**Female**									
	t	-.552	-.922	-.440	-.844	-1.061	-1.375	-1.533	-1.395
	p_{uncorr}	.587	.351	.651	.392	.287	.171	.119	.165
	p_{FDR}	.651	.522	.651	.522	.522	.457	.457	.457
	d	-.276	-.462	-.220	-.422	-.531	-.688	-.768	-.698
**Male > Female**									
	t	-.268	-.637	-.171	.385	-.111	.403	.608	.773
	p_{uncorr}	.796	.515	.863	.701	.913	.685	.542	.446
	p_{FDR}	.913	.913	.913	.913	.913	.913	.913	.913
	d	-.128	-.304	-.082	.184	-.053	.192	.290	.369
**Female > Male**									
	t	.268	.637	.171	-.385	.111	-.403	-.608	-.773
	p_{uncorr}	.796	.515	.863	.701	.913	.685	.542	.446
	p_{FDR}	.913	.913	.913	.913	.913	.913	.913	.913
	d	.128	.304	.082	-.184	.053	-.192	-.290	-.369

p_{uncorr}: uncorrected p-values; p_{FDR}: FDR-corrected p-values; *d*: Cohen's *d*; rACC: rostral anterior cingulate cortex; cACC: caudal ACC; PCC: posterior cingulate cortex; lOFC: lateral orbitofrontal cortex; mOFC: medial OFC. Cortisol contrast represents a sex-independent cortisol increase using the whole sample. Significant results are given in bold.

Table S6. Results of PALM calculations for cortical thickness of the left hemisphere.

Contrast		rACC	cACC	PCC	Parahippocampus	lOFC	mOFC	Insula	Precuneus
**Cortisol**									
	t	-1.073	-.559	.035	-.303	**2.218**	.738	-.059	.318
	p_{uncorr}	.283	.578	.974	.764	**0.028**	.463	.952	.744
	p_{FDR}	.974	.974	.974	.974	0.226	.974	.974	.974
	d	-.439	-.229	.014	-.124	0.908	.302	-.024	.130
**Male**									
	t	-.108	-.759	-.381	-.548	1.287	.059	1.168	-.135
	p_{uncorr}	.913	.447	.704	.576	0.203	.955	.242	.892
	p_{FDR}	.955	.955	.955	.955	0.955	.955	.955	.955
	d	-.052	-.366	-.184	-.264	0.621	.029	.564	-.065
**Female**									
	t	-1.243	-.140	.318	.023	1.801	.866	-.917	.488
	p_{uncorr}	.217	.887	.753	.981	0.078	.390	.356	.624
	p_{FDR}	.780	.981	.981	.981	0.627	.780	.780	.981
	d	-.622	-.070	.159	.011	0.902	.433	-.459	.245
**Male > Female**									
	t	.942	-.331	-.480	-.339	-0.705	-.666	1.425	-.438
	p_{uncorr}	.346	.735	.633	.730	0.488	.503	.152	.639
	p_{FDR}	.735	.735	.735	.735	0.735	.735	.735	.735
	d	.450	-.158	-.229	-.162	-0.336	-.318	.680	-.226
**Female > Male**									
	t	-.942	.331	.480	.339	0.705	.666	-1.425	.438
	p_{uncorr}	.346	.735	.633	.730	0.488	.503	.152	.639
	p_{FDR}	.735	.735	.735	.735	0.735	.735	.735	.735
	d	-.450	.158	.229	.162	0.336	.318	-.680	.226

p_{uncorr}: uncorrected p-values; p_{FDR}: FDR-corrected p-values; d: Cohen's d; rACC: rostral anterior cingulate cortex; cACC: caudal ACC; PCC: posterior cingulate cortex; lOFC: lateral orbitofrontal cortex; mOFC: medial OFC. Cortisol contrast represents a sex-independent cortisol increase using the whole sample. Significant results are given in bold.

Table S7. Results of PALM calculations for cortical thickness of the right hemisphere.

Contrast	rACC	cACC	PCC	Parahippocampus	lOFC	mOFC	Insula	Precuneus
**Cortisol**								
t	.142	**2.037**	1.051	-.953	1.131	1.202	-.048	-.126
p_{uncorr}	.886	**.046**	.301	.344	.263	.229	.965	.902
p_{FDR}	.965	.366	.550	.550	.550	.550	.965	.965
d	.058	.834	.430	-.390	.463	.492	-.020	-.052
**Male**								
t	.791	.849	.854	-.450	1.077	.366	.446	-.336
p_{uncorr}	.420	.397	.396	.649	.277	.715	.652	.773
p_{FDR}	.733	.733	.733	.733	.733	.733	.733	.733
d	.382	.410	.412	-.217	.519	.177	.215	-.162
**Female**								
t	-.397	1.894	.677	-.848	.615	1.216	-.382	.088
p_{uncorr}	.687	.060	.506	.390	.539	.226	.704	.929
p_{FDR}	.805	.478	.805	.805	.805	.805	.805	.929
d	-.199	.948	.339	-.425	.308	.609	-.191	.044
**Male > Female**								
t	.783	-1.036	-.049	.424	.132	-.770	.570	-.267
p_{uncorr}	.433	.296	.960	.667	.895	.444	.565	.792
p_{FDR}	.960	.960	.960	.960	.960	.960	.960	.960
d	.374	-.495	-.023	.202	.063	-.367	.272	-.128
**Female > Male**								
t	-.783	1.036	.049	-.424	-.132	.770	-.570	.267
p_{uncorr}	.433	.296	.960	.667	.895	.444	.565	.792
p_{FDR}	.960	.960	.960	.960	.960	.960	.960	.960
d	-.374	.495	.023	-.202	-.063	.367	-.272	.128

p_{uncorr}: uncorrected *p*-values; p_{FDR}: FDR-corrected *p*-values; *d*: Cohen's *d*; rACC: rostral anterior cingulate cortex; cACC: caudal ACC; PCC: posterior cingulate cortex; lOFC: lateral orbitofrontal cortex; mOFC: medial OFC. Cortisol contrast represents a sex-independent cortisol increase using the whole sample. Significant results are given in bold.

Table S8. Results of PALM calculations for subcortical volume of the left hemisphere.

Contrast		Thalamus	Ncl. Caudatus	Ncl. Accumbens	Putamen	Hippocampus	Amygdala
**Cortisol**							
	t	-.489	1.530	.416	.257	-.137	.332
	p_{uncorr}	.628	.126	.680	.797	.889	.744
	p_{FDR}	.889	.758	.889	.889	.889	.889
	d	-.197	.616	.168	.103	-.055	.134
**Male**							
	t	-.383	1.926	.273	.384	.254	.122
	p_{uncorr}	.703	.054	.778	.705	.796	.899
	p_{FDR}	.899	.323	.899	.899	.899	.899
	d	-.184	.928	.132	.185	.123	.059
**Female**							
	t	-.328	.500	.317	.040	-.353	.322
	p_{uncorr}	.740	.618	.759	.969	.726	.753
	p_{FDR}	.911	.911	.911	.969	.911	.911
	d	-.163	.248	.157	.020	-.175	.160
**Male > Female**							
	t	.042	.725	-.097	.193	.436	-.191
	p_{uncorr}	.969	.476	.919	.848	.664	.849
	p_{FDR}	.969	.969	.969	.969	.969	.969
	d	.020	.346	-.046	.092	.208	-.091
**Female > Male**							
	t	-.042	-.725	.097	-.193	-.436	.191
	p_{uncorr}	.969	.476	.919	.848	.664	.849
	p_{FDR}	.969	.969	.969	.969	.969	.969
	d	-.020	-.346	.046	-.092	-.208	.091

p_{uncorr}: uncorrected p-values; p_{FDR}: FDR-corrected p-values; d: Cohen's d. Cortisol contrast represents a sex-independent cortisol increase using the whole sample. Significant results are given in bold.

Table S9. Results of PALM calculations for subcortical volume of the right hemisphere.

Contrast		Thalamus	Ncl. Caudatus	Ncl. Accumbens	Putamen	Hippocampus	Amygdala
**Cortisol**							
	t	1.023	1.861	.467	-.289	-.440	-.750
	p_{uncorr}	.300	.065	.652	.768	.655	.448
	p_{FDR}	.768	.392	.768	.768	.768	.768
	d	.412	.750	.188	-.117	-.177	-.302
**Male**							
	t	.017	**2.303**	.468	.871	.242	.588
	p_{uncorr}	.986	**.023**	.639	.391	.810	.561
	p_{FDR}	.986	.137	.958	.958	.972	.958
	d	.008	1.110	.225	.420	.116	.283
**Female**							
	t	1.252	.637	.239	-.987	-.718	-1.352
	p_{uncorr}	.203	.527	.807	.323	.465	.175
	p_{FDR}	.610	.632	.807	.632	.632	.610
	d	.621	.316	.119	-.490	-.356	-.671
**Male > Female**							
	t	-1.007	.835	.081	1.312	.725	1.443
	p_{uncorr}	.319	.412	.938	.192	.474	.148
	p_{FDR}	.569	.569	.938	.569	.569	.569
	d	-.481	.399	.038	.627	.346	.689
**Female > Male**							
	t	1.007	-.835	-.081	-1.312	-.725	-1.443
	p_{uncorr}	.319	.412	.938	.192	.474	.148
	p_{FDR}	.569	.569	.938	.569	.569	.569
	d	.481	-.399	-.038	-.627	-.346	-.689

p_{uncorr}: uncorrected p-values; p_{FDR}: FDR-corrected p-values; d: Cohen's d. Cortisol contrast represents a sex-independent cortisol increase using the whole sample. Significant results are given in bold.

Table S10. Cortisol increase prediction scores using different brain measure and confound correction settings.

Brain Measures	Metric		Metric	
	r	$p_{permutation}$	*EV*	$p_{permutation}$
Surface area, Thickness, Volume	.124	.039	.004	.028
Thickness, Volume	.029	.383	-.006	.685

r, Pearson's correlation coefficient between true and predicted cortisol increase values; $P_{permutation}$, *p*-values derived from permutation testing, EV, Explained Variance.

Supplementary Figures

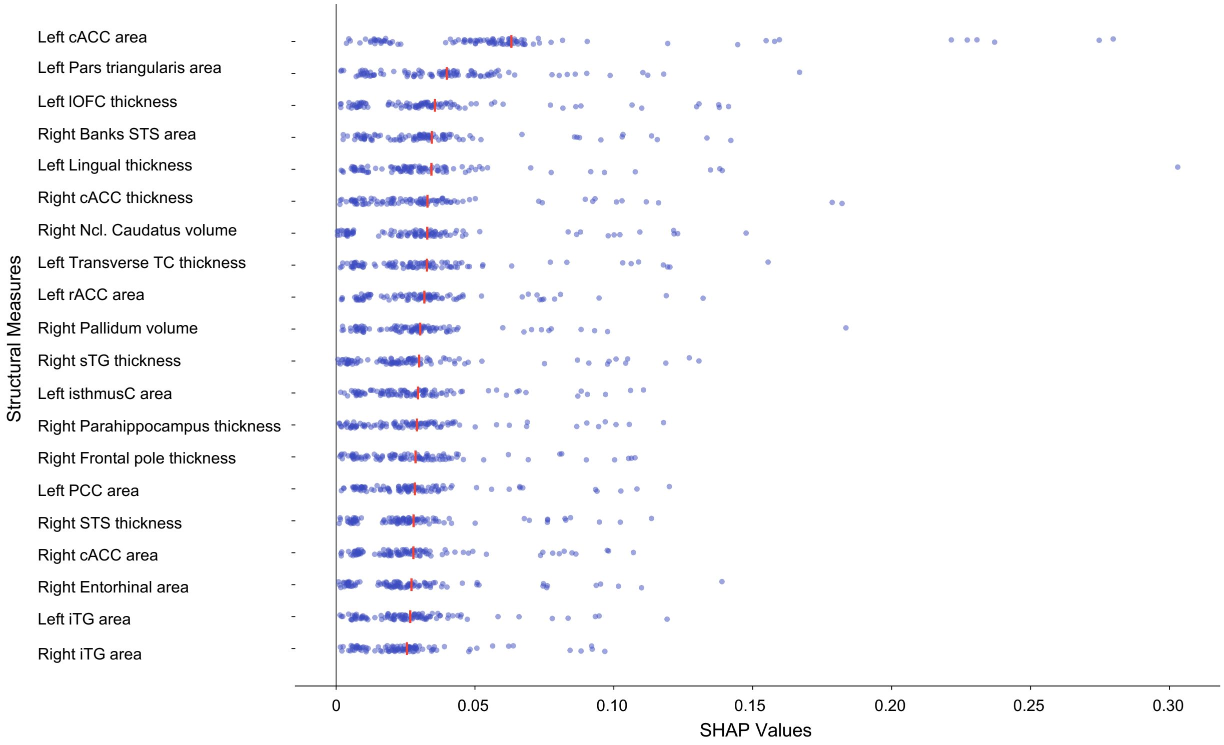

Figure S1. The most informative structural measures for predicting cortisol increase, as measured by SHAP values derived from a ridge regression model within each cross-validation fold. The absolute extent of feature contribution is depicted by SHAP values, with each point representing the median of the absolute SHAP values within each cross-validation fold. Mean SHAP values of each brain measure are indicated by a red line. The brain regions are denoted as follows: cACC, caudal anterior cingulate cortex; STS, superior temporal sulcus; lOFC, lateral orbitofrontal cortex; TC, temporal cortex; Ncl. Caudatus, nucleus caudatus; isthmusC, isthmus cingulate; sTG, superior temporal gyrus; PCC, posterior cingulate cortex; iTG, inferior temporal gyrus.

Supplementary References

Lundberg, S., & Lee, S.-I. (2017). *A Unified Approach to Interpreting Model Predictions* (Version 2). arXiv. https://doi.org/10.48550/ARXIV.1705.07874
